# Induction of Apoptosis by the Nonstructural Protein 4 and 10 of Porcine Reproductive and Respiratory Syndrome Virus

**DOI:** 10.1371/journal.pone.0156518

**Published:** 2016-06-16

**Authors:** Shuaizhen Yuan, Ning Zhang, Lei Xu, Lei Zhou, Xinna Ge, Xin Guo, Hanchun Yang

**Affiliations:** 1 Key Laboratory of Animal Epidemiology and Zoonosis of Ministry of Agriculture, College of Veterinary Medicine, China Agricultural University, Beijing, People’s Republic of China; 2 State Key Laboratory of Agrobiotechnology, China Agricultural University, Beijing, People’s Republic of China; Institute of Biochemistry and Biotechnology, TAIWAN

## Abstract

Infection by most viruses triggers apoptosis in host cells, and viruses manipulate this cell response to promote viral replication, virus spread, and cell killing. Porcine reproductive and respiratory syndrome virus (PRRSV) has been shown to induce apoptosis both *in vitro* and *in vivo*, while the regulatory roles of PRRSV-encoded products in apoptosis are not fully understood. In the present study, we first showed a biphasic apoptosis regulation by a highly pathogenic PRRSV strain JXwn06. It was indicated that PRRSV infection delays apoptosis at early infection but activates apoptosis at late infection in MARC-145 cells. In PRRSV-infected MARC-145 cells, procaspase-8, -9 and -12 were activated at late infection, demonstrating the involvements of death receptor pathway, mitochondrial pathway and endoplasmic reticulum (ER) stress pathway in inducing apoptosis. PRRSV was also shown to induce a similar apoptosis process in pulmonary alveolar macrophages (PAMs) with an early initiation. Next, the PRRSV-encoded apoptosis inducers were screened, indicating that the nonstructural protein (Nsp) 4 and Nsp10 of PRRSV are pro-apoptotic. In the presence of Nsp4, it was confirmed that procaspase-8, -9 and -12 were cleaved, and Nsp4 facilitates the cleavage of procaspase-9 by activating B-cell lymphoma 2 interacting mediator of cell death (Bim), a pro-apoptotic protein. In addition, Nsp4 was shown to induce the degradation of an anti-apoptotic protein, B-cell lymphoma-extra large (Bcl-xL). Nsp10 was shown to activate procaspase-8 and -9 but procaspase-12 and to upregulate the expression of BH3-only pro-apoptotic protein BH3 interacting-domain death agonist (Bid) and its active form, truncated Bid (tBid). Clearly, the participation of both activated caspase-8 and Bid is required for Nsp10-induced apoptosis, indicating a crosstalk between extrinsic- and mitochondria-dependent pathways. Together, our findings suggest that PRRSV infection regulates apoptosis in a two-phase manner and activates all three apoptotic pathways; the Nsp4 and Nsp10 of PRRSV function as apoptosis inducers with different molecular basis.

## Introduction

Apoptosis is a continually occurring, tightly regulated process in multicellular organisms in response to a variety of stimuli, including virus infection [1,2]. In general, apoptotic pathways can be divided into two nonexclusive signaling cascades: extrinsic pathway and intrinsic pathway [3]. The latter can be further separated into mitochondria-dependent and endoplasmic reticulum (ER) stress-dependent pathways. Ensured apoptotic signal transductions activate executioners like caspase-3 and initiate the execution phase of apoptosis [4]. Tremendous studies have determined a large group of proteins manipulating apoptosis. Bax and Bak are pro-apoptotic molecules which activate caspase-9 [5]. In addition, BH3-domain-only Bcl-2 members, Bid, Bim, and Puma also participate in Bax- and Bak-induced apoptosis [6]. Bcl-2 and Bcl-xL possess anti-apoptotic functions and both of them block apoptosis by inhibiting homo-oligomerization and activation of Bax and Bak [7].

Porcine reproductive and respiratory syndrome (PRRS) is characterized by reproductive failure in sows and respiratory disorders in growing pigs, as well as high mortality in piglets, causing huge economic losses to the swine industry worldwide [8–10]. PRRS virus (PRRSV), the etiological agent, is classified into the family *Arteriviridae* in the order *Nidovirales* [11]. To date, all PRRSV isolates characterized worldwide can be grouped into two distinct genotypes, the European genotype 1 and North American genotype 2 [12–14]. PRRSV is an enveloped RNA virus, with at least 11 open reading frames (ORFs) within its positive-sense RNA genome, which encode nonstructural proteins (Nsps) and structural proteins [15–18]. Ribosomal frame-shifting (RF) signal mediates the synthesis of two polyproteins, pp1a and pp1ab, which is cleaved into 14 Nsps [19–21]. The Nsps of PRRSV have been shown to be essential for virus replication and genomic transcription, viral pathogenesis and virulence [22,23]. A growing number of studies have also indicated that the Nsps of PRRSV play important roles in modulating innate immune responses of host [24–26].

Previous studies have determined the regulatory roles of PRRSV in apoptosis that is important for the pathogenesis of PRRSV [27–32]. PRRSV-infected cells have been shown to present evident activation of caspase-8 and caspase-9, representing the activation of extrinsic pathway and intrinsic pathway, respectively [33]. Moreover, the Bid truncated by PRRSV-activated caspase-8 subsequently induces caspase-9-depedndent apoptosis, suggesting a crosstalk between these two pathways [33]. Besides Bid, other Bcl-2 family members such as Bax, Bcl-2 and Bcl-xL participate in PRRSV-induced apoptosis as well [33,34]. PRRSV also regulates apoptosis by manipulating multiple signaling pathways [34–36]. At the late infection, PRRSV activates c-Jun N-terminal kinase (JNK) pathway but suppresses phosphatidylinositol-3-kinase (PI3K)-dependent Akt pathway (PI3K/Akt), to promote PRRSV-induced apoptosis [34]. On the contrary, PRRSV activates PI3K/Akt and p53 pathways at the early stage of infection, which counteracts PRRSV-mediated apoptosis [35]. PRRSV-induced apoptosis has been suggested to be viral replication-dependent; hence the product (s) encoded by viral genes may play pro-apoptotic roles [29,30,32,37]. The pro-apoptotic function of PRRSV GP5 has been recognized previously [38–40], while recent studies have showed clearly that the GP5 is dispensable for apoptosis [41,42].

Highly pathogenic PRRSV (HP-PRRSV) belonging to type 2 emerged in China in 2006 [43], and its prevalence has caused inestimable loss to the Chinese swine industry [44]. Although the Nsp9- and Nsp10-coding regions together of HP-PRRSV have been shown to play critical roles in its replication efficiency and fatal virulence for piglets [23], the precise mechanism in relation to its pathogenicity, particularly the roles of its Nsps in pathogenesis such as apoptosis, replication regulation and immunomodulation, is yet to be clarifed. In the present study, we employed a strain of HP-PRRSV (JXwn06) to focus on investigating the PRRSV-induced apoptosis process and its involved apoptotic pathways, and screening PRRSV-encoded apoptotic inducers among the Nsps, in an attempt to provide novel insights into the pathogenesis of HP-PRRSV.

## Materials and Methods

### Cells, Virus and Infection

The African green monkey kidney epithelial cell line MARC-145 cells [45] and human embryonic kidney HEK-293FT cells (Cell Resource Center, Institute of Basic Medical Science, CAMS/PUMC) [46] were cultured with Dulbecco’s modified Eagle medium (DMEM) (Fisher Scientific, Waltham, MA), supplemented with 10% heat-inactivated fetal bovine serum (FBS, Hyclone Laboratories, Inc., South Logan, UT) in a humidified incubator with 5% CO_2_ at 37°C. PRIM-1640 medium (Fisher Scientific, Waltham, MA) were used to cultivate porcine pulmonary alveolar macrophages (PAMs). A strain of HP-PRRSV JXwn06 was used in this study [47]. For virus infection, MARC-145 cells were grown to approximately 90% confluency, and PAMs were prepared as described previously [48], then cells were infected at multiplicity of infection (MOI) of 1 and maintained with respective medium containing 5% FBS at 37°C until collection.

### Antibodies and Chemicals

An anti-PRRSV-Nsp1β mouse monoclonal antibody (MAb) that specifically interacts with the Nsp1β of type 2 PRRSV was generated in our laboratory. Anti-PARP polyclonal antibody (PAb) was purchased from Abcam (Cambridge, UK). Anti-caspase-8 PAb, anti-caspase-9 PAb, anti-Bim PAb, anti-Bid PAb, and anti-Bcl-xL PAb were purchased from Cell Signaling Technology (Beverly, MA). Anti-caspase-12 PAb, anti-HA MAb, and anti-β-actin MAb were purchased from Sigma-Aldrich (St Louis, MO). Anti-Bax MAb, anti-Bcl-2 PAb, and anti-caspase-3 p17 PAb were purchased from Santa Cruz Biotechnologies Inc (Santa Cruz, CA). Anti-Cytochrome *c* MAb was purchased from Calbiochem (San Diego, CA). Anti-GFP PAb and anti-VDAC1 PAb were purchased from Proteintech (Chicago, IL). DAPI (4’, 6-diamidino-2-phenylindol) and PMSF (phenylmethylsulfonyl fluoride) were purchase from Beyotime **(**Nanjing, China).

### Drug Treatment

Apoptosis inducers, Thapsigargin (TG; Sigma-Aldrich, St Louis, MO) and staurosporine (STS; Merck, Darmstadt, Germany), were diluted in dimethyl sulfoxide (DMSO). These two drugs were added in cell cultures at a final concentration of 100 nM for 4 h, respectively, and then cells were harvested for subsequent Western blot analysis. To inhibit caspase-8 activation, MARC-145 cells were treated with Z-IETD-FMK (Sigma) for 30 min at a final concentration of 30 μM.

### Plasmid Construction and DNA Transfection

Plasmids containing full-length of individual Nsp gene including Nsp1α, Nsp1β, Nsp2-Nsp5, Nsp7, Nsp8-Nsp12 with a HA-tag were constructed and conserved in our laboratory [49]. The full sequence of each of the Nsp genes was amplified by PCR using the infectious clone pWSK-JXwn as a template [47]. The PCR fragments were cloned into the pCMV-HA (Clontech) mammalian expression vector. To construct lentiviral plasmids, individual Nsp gene was cloned into the pWPXL vector, separately. A C’-terminal GFP tag was fused to each DNA construct.

Transfection was conducted using Lipofectamine™ LTX with Plus reagent according to the manufacturer’s instruction (Fisher Scientific, Waltham, MA). MARC-145 cells were plated in 6-well plates and grown to 60% confluency. Transfection mix containing 2.5 μg of plasmid DNA, 2.5 μL of plus reagent and 10 μL of Lipofectamine LTX in Opti-MEM I (Fisher Scientific, Waltham, MA) was incubated at room temperature (RT) for 25 min and added to each well. Cells were cultured for 24 h for protein expression.

### Lentiviral Transduction

To package individual Nsp gene into lentivirus particles, HEK-293FT cells were co-transfected with DNA constructs carrying individual Nsp gene of PRRSV, separately, along with pMD2.G and psPAX2 at a ratio of 2:2:1 using FuGene transfection reagent (Promega, WI). Supernatants containing lentivirus were harvested twice at 48 h and 72 h post-transfection, respectively, and filtered with Centrifugal Filter (Millipore, Cat No. UFC910096) at 4°C with subsequent resuspension using lentiviral store solution. The titers of lentivirus particles were determined on MARC-145 cells through serial dilution method. Then, lentiviruses carrying individual packaged Nsp gene were used to infect MARC-145 cells at MOI of 50 to 100. Subsequently, infected cells were passaged once and maintained for 24 h before collection. Considering cytotoxic effect of PRRSV Nsps, lentivirus-infected cells were not used to develop stable cell lines with G418 selection, but were used directly for Western blot analysis. The expression of individual Nsp in MARC-145 cells was confirmed under fluorescence microscope and meanwhile their expression efficiency with above 60% was examined using flow cytometry.

### Immunofluorescence Assay (IFA)

Cells were seeded on fibronectin-coated cover slips and grown to 80% confluency. And then cells were transfected with individual plasmids for 24 h. After washing with phosphate-buffered saline (PBS), cells were fixed with 4% paraformaldehyde for 30 min at RT in PBS, and then permeabilized using 0.1% Triton X-100 for 10 min at RT. After blocking with 1% BSA in PBS for 30 min, cells were incubated with primary antibody in PBS containing 1% BSA for 2 h followed by incubation with FITC-conjugated (ZSBio, Beijing, China) or TRICT-conjugated (ZSBio) secondary antibodies for 1 h at RT. The staining of the nucleus was performed with DAPI for 3 min at RT. After washing with PBS, coverslips were mounted onto microscope slides using anti-fade mounting medium (Beyotime), and visualized under the FLUOVIEW FV1000 confocal scanning laser microscopy system (Olympus, Tokyo, Japan). Images were processed with NIH Image/J for analyzing florescence intensity (FI).

### Western Blot Analysis

Cells were harvested using RIPA lysis buffer (Beyotime) supplemented with 0.1% PMSF (Beyotime). Protein concentration was determined by BCA method. Cell lysates were centrifuged at 20,000× g at 4°C for 10 min, and supernatants were resolved by 10% or 12% Sodium dodecyl Sulfate (SDS)-poly-acrylamide gel electrophoresis (SDS-PAGE) and transferred to polyvinylidene fluoride (PVDF) membrane (Millipore, Billerica, MA). After blocking with 5% skim milk powder in TBS-T (10 mM Tris–HCl [pH 8.0], 150 mM NaCl, 1% Tween 20), membranes were incubated with primary antibodies dissolved in TBS-T containing 5% skim milk powder for 1 h at RT followed by washing and incubation with horseradish peroxidase (HRP)-conjugated secondary antibodies (ZSBio) for 1 h at RT. After three washes with TBS-T, proteins were visualized using the enhanced chemiluminescence Western blot kit (CWBIO, Beijing, China). Images were obtained using FluorE system (ProteinSimple, Santa Clara, CA) and densitometry was performed with the Alphaview v3.0 software.

### Assessment of Apoptosis

The release of cytochrome *c*, activation of caspase-3, and the cleavage of PARP were employed to assess PRRSV-induced apoptosis. To examine the cytosolic cytochrome *c*, the cytosol and mitochondria of PRRSV-infected MARC-145 cells were fractionated using the cytosol/mitochondrial fractionation kit according to the manufacturer’s protocol (Calbiochem). Flow cytometry was used to detect activated caspase-3. Briefly, infected or uninfected cells were fixed with 4% paraformaldehyde and incubated with PE-conjugated caspase-3 antibody (BD Pharmingen, San Diego, CA) for 40 min. The percentages of caspase-3-activated cells were counted with Coulter Cell Lab QuantaSC flow cytometer (Beckman Coulter, Fullerton, CA). PARP cleavage was determined by Western blot using anti-PARP PAb (1:400, Abcam).

### RNA Interference

The small interfering RNAs (siRNAs) against monkey Bid (5’-GAGGAGCUUAGCCAGAAAUTT-3’) were purchased from GenePharma (Shanghai, China). A nonspecific siRNA sequence (5’-UUCUCCGAACGUGUCACGUTT-3’) was used as a negative control. MARC-145 cells were transfected with 30 nM of each indicated siRNAs using Lipofectamine™ RNAiMAX (Fisher Scientific, Waltham, MA) according to the manufacturer’s protocol.

### Cell Viability Assay

Cell viability assay was conducted to determine the viability of MARC-145 cells following the treatment of apoptosis inducers, STS and TG. Cells were plated in 96-well tissue culture plates at a density of 5 × 10^4^ cells/well and cultured for 24 h, followed by exposure to drugs for 24 h. After washed with PBS, CellTiter 96 Aqueous One solution (Promega, Madison, WI) was inoculated with following incubation for 4 h at 37°C. Absorbance was measured at 490 nm. Each condition was replicated for eight times.

### Statistical Analysis

The data represents the mean values and SDs from three independent experiments. All statistical analyses were performed using Graphpad Prism version 5.0 software. Statistical comparisons were performed using One-way ANOVA with Bonferroni correction. Significant differences (*P* < 0.05) are denoted in figures by asterisks.

## Results

### Biphasic Function of PRRSV in Apoptosis

**Fig 1:**
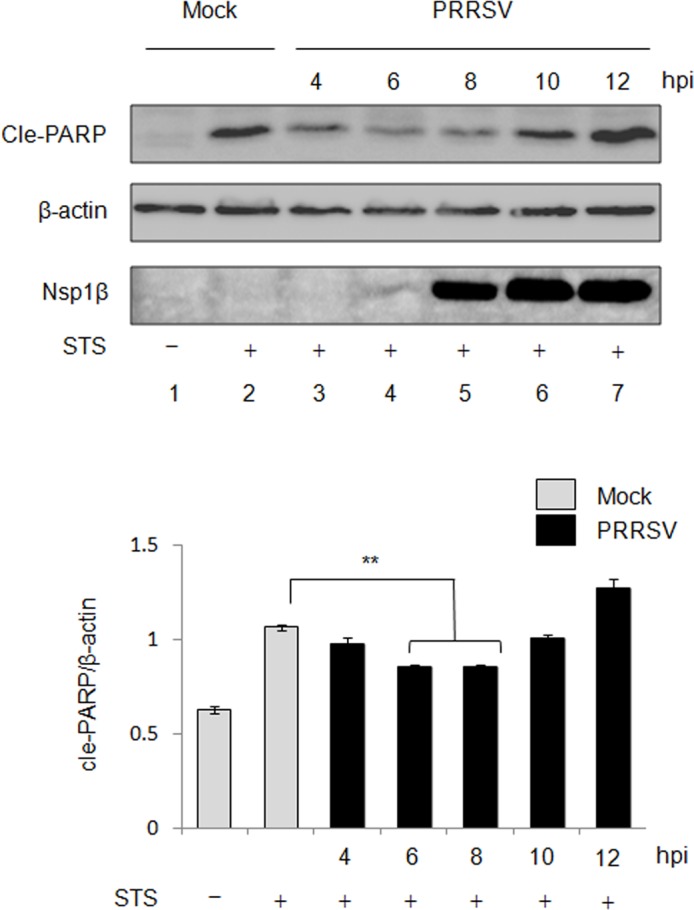
Apoptosis inhibition by PRRSV at the early stage of infection. MARC-145 cells were infected with PRRSV JXwn06 at an MOI of 1 for 4 h, 6 h, 8 h, 10 h, and 12 h. Chemical apoptosis inducer STS was added at the final concentration of 100 nM for 4 h before harvesting cells. Total cell lysates were then subjected to Western blot using anti-β-actin, anti-PARP, and anti-Nsp1β antibodies. β-actin was served as loading control. Relative level of cleaved (cle)-PARP. Densitometry analysis showed the relative cle-PARP level after normalization with β-actin. Significant differences of relative cle-PARP level between STS-treated non-infected cells and each of PRRSV-infected cells are indicated (* *P*<0.05; ** *P*<0.01; *** *P*<0.001). Data are means±standard deviations (error bars) from three independent trials.

The dichotomous role of PRRSV in modulating apoptosis has been determined using a strain of type 1 PRRSV previously. PRRSV stimulates anti-apoptotic pathways early in infection while induces apoptosis late in infection [37]. Because of the genetic difference between type 1 and type 2 PRRSV, it is of interest to determine if modulatory roles in apoptosis are identical between the two types of PRRSV [13,14]. A strain of type 2 PRRSV JXwn06 was used to infect MARC-145 cells in this study to examine whether type 2 PRRSV suppress apoptosis at the early stage of infection. MARC-145 cells were infected with PRRSV at an MOI of 1 for 4 h, 6 h, 8 h, 10 h, and 12 h, separately. PRRSV-infected cells were incubated with STS, a chemical apoptosis inducer, for 4 h before terminating the infection. Cell viability assay showed that the STS treatment had no influence on the viability of MARC-145 cells (S1 Fig). Total cells lysates were then subjected to Western blot to examine the cleavage of PARP as a reliable hallmark of apoptosis induction[50]. As shown in Fig 1, in comparison with STS-treated uninfected cells, the PARP cleavages were less evident from 4 h post-infection (pi) to 8 h pi in PRRSV-infected MARC-145 cells, and the reduction of PARP cleavage were significant at 6 h pi and 8 h pi (*P*<0.01), while the level of cleaved PARP was increased at 12 h pi. PRRSV Nsp1β was examined to monitor viral replication, and newly synthesized Nsp1β appeared as early as 6 h pi. These data suggested that type 2 PRRSV suppressed apoptosis at early infection, which is consistent to the type 1 PRRSV.

**Fig 2:**
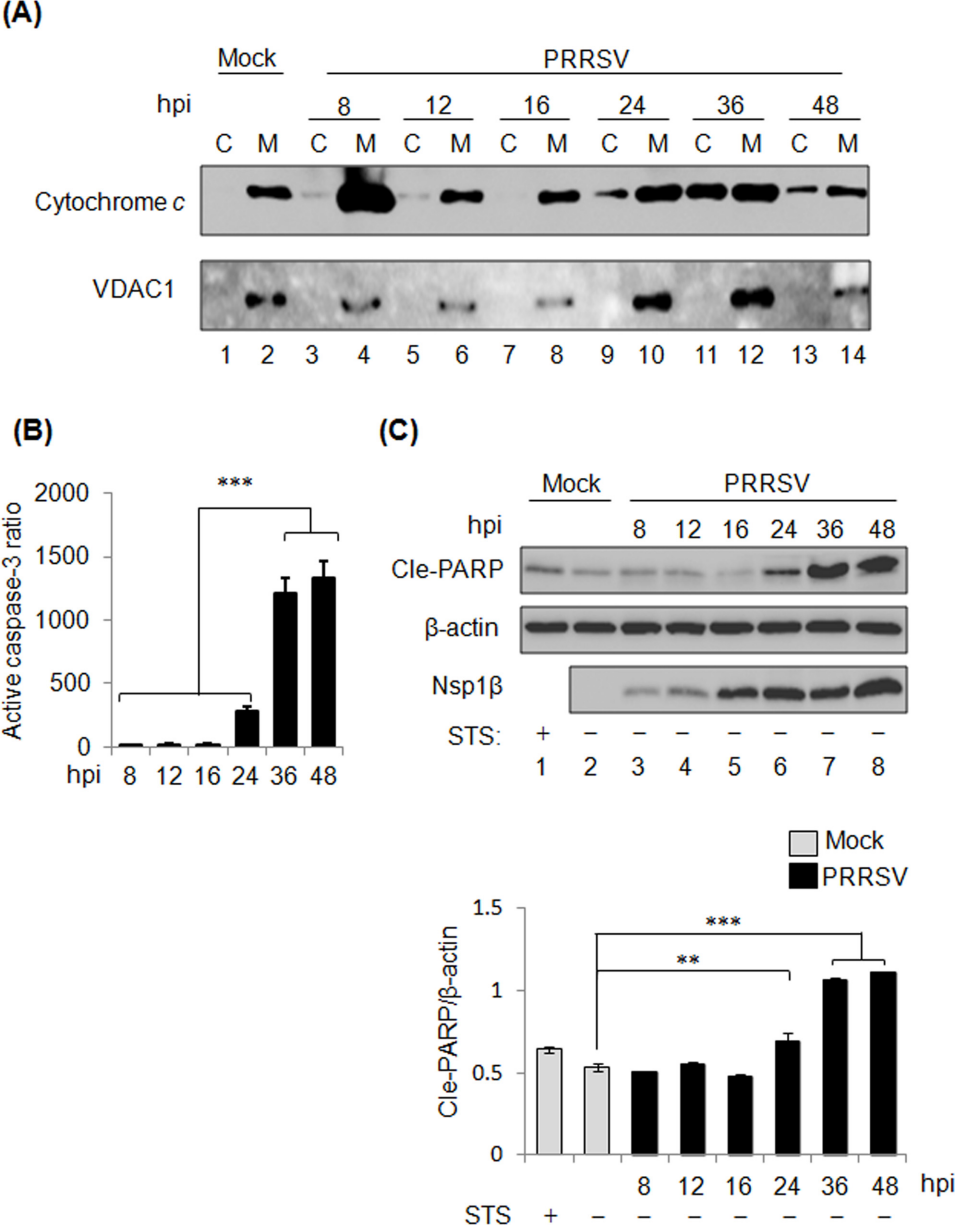
PRRSV-induced apoptosis at the late stage of infection. (A) The release of cytochrome *c* in PRRSV-infected MARC-145 cells. PRRSV-infected MARC-145 cells were subjected to mitochondrial/cytosolic fractionation at 8 h, 12 h, 16 h, 24 h, 36 h and 48 h post-infection (pi). Cytosolic and mitochondrial fractions were subjected to Western blot using anti-cytochrome *c* and anti-VDAC1 antibodies. VDAC1 was selected as a mitochondrial protein marker. (B) PRRSV-infected MARC-145 cells were harvested at the indicated time points, and then fixed and assessed by flow cytometry analysis using direct staining with PE-conjugated active caspase-3 antibody. Active caspase-3 ratio was calculated by normalizing the percentage of PRRSV-infected caspase-3-activated cells with the percentage of uninfected cells. Data are means±standard deviations (error bars) from three independent trials. Significant differences of active caspase-3 ratio between early and late infection groups are indicated (*P<0.05; **P<0.01; ***P<0.001). (C) PRRSV-induced PARP cleavage. STS-treated cells were used as a positive control for PARP cleavage. Cle-PARP in PRRSV-infected cells was examined by Western blot. The intensity of cle-PARP was calculated by densitometry analysis. Relative level of cle-PARP was normalized with β-actin. Significant differences of cle-PARP relative level between non-infected and infected groups are indicated (*P<0.05; **P<0.01; *** *P*<0.001).

The apoptosis induction by type 2 PRRSV were investigated in MARC-145 cells, and examinations on the hallmarks of apoptosis were conducted, including the release of cytochrome *c*, activation of caspase-3, and PARP cleavage (Fig 2). The release of cytochrome *c* from mitochondria to cytosol indicates the activation of intrinsic apoptotic pathway [51]. As shown in Fig 2A, cytosolic cytochrome *c* appeared at 24 h pi and reached a peak at 36 h pi with a subsequent decrease at 48 h pi, while VDAC1 as mitochondrial protein marker remained in its subcellular compartments, suggesting that PRRSV induces apoptosis at late infection. The activation of caspase-3, an apoptosis executioner, was analyzed by using quantitative flow cytometric assay, and the active caspase-3 ratio representing the normalized percentage of caspase-3-activated cells was calculated (Fig 2B and S1 Table). At 24 h pi, active caspase-3 ratio started to increase compared to the ratios at 8 h pi, 12 h pi, and 16 h pi, whereas active caspase-3 ratio was significantly augmented at 36 h pi and 48 h pi (*P*<0.001) (Fig 2B), in the comparison with other time points. As shown in Fig 2C, PRRSV-mediated PARP cleavage initiated at 24 h pi, and the cleavage became evident at 36 h pi and 48 h pi. Based on the densitometry analysis, the cleavage of PARP was significant from 24 h pi to 48 h pi compared to the uninfected cells without STS (*P*<0.05 or 0.001). Taken together, our results demonstrated that type 2 PRRSV infection initiated apoptosis at 24 h pi and maintained this process at the late stage of infection, and type 2 PRRSV possessed identical dichotomous role in modulating apoptosis to type 1 PRRSV.

### Activated Apoptosis Pathways in PRRSV-Infected MARC-145 Cells

**Fig 3:**
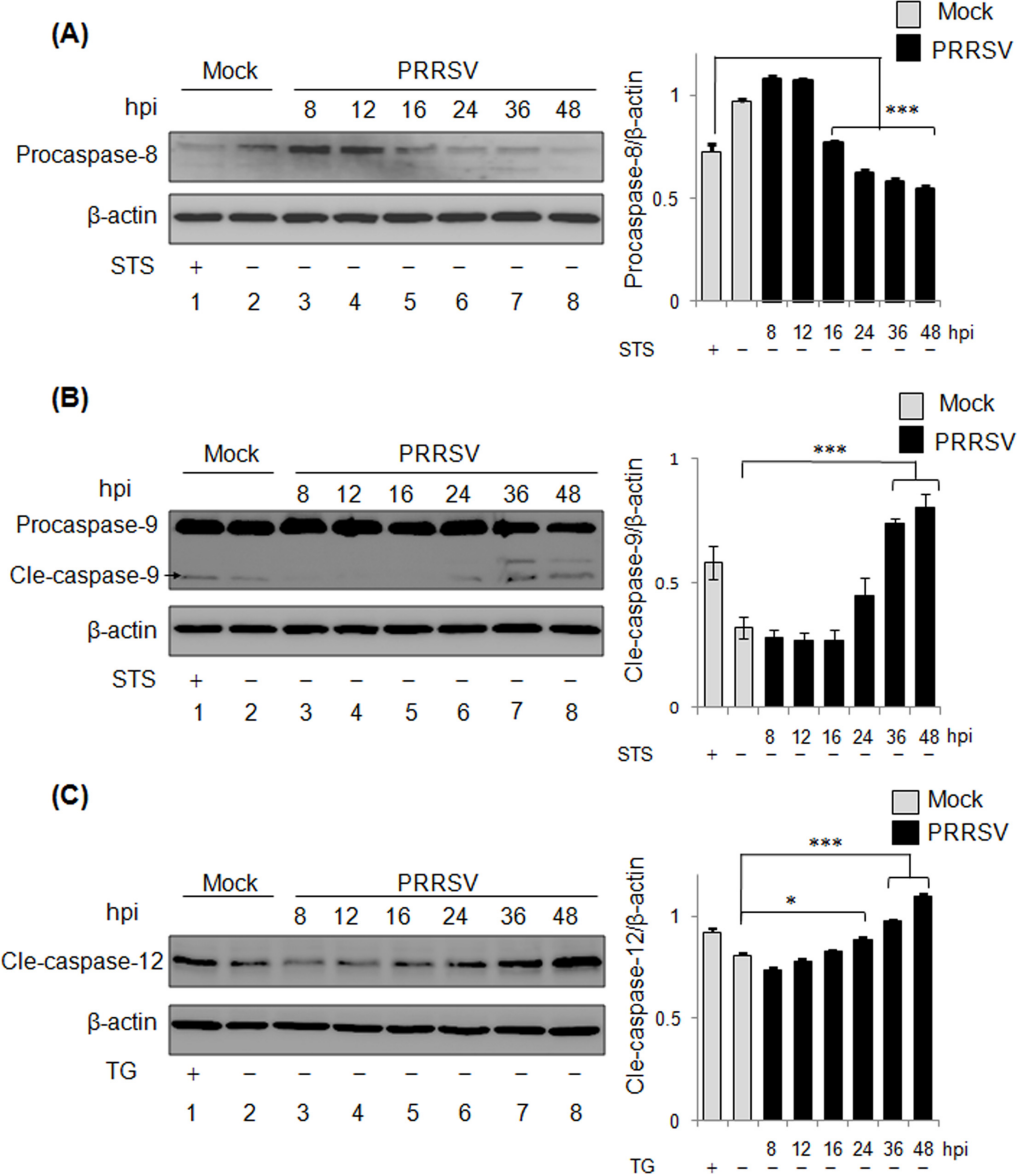
Activations of caspase-8, -9 and -12 in PRRSV-infected MARC-145 cells. MARC-145 cells were infected PRRSV for 8 h, 12 h, 16 h, 24 h, 36 h, and 48 h. STS or TG treatments were used to induce the activation of caspase-8 (A) and -9 (B), and caspase-12 (C), respectively. The activation of caspase-8, -9 and -12 was examined by Western blot using anti-caspase-8 (A), anti-caspase-9 (B) and anti-cleaved (cle) caspase-12 (C) antibodies, respectively. β-actin was served as a protein loading control. The intensity of procaspase-8, cle-caspase-9, and cle-caspase-12 was normalized with the one of β-actin to obtain their relative levels. Significant differences of relative levels between control group and infected groups are indicated (* *P*<0.05; ** *P*<0.01; *** *P*<0.001).

Since PRRSV was shown to induce apoptosis of MARC-145 cells at late infection, we further clarified the involved apoptosis pathways in PRRSV-infected cell. Cell viability assay showed that the STS or TG treatment did not impact the viability of MARC-145 cells (S1 Fig). The activations of caspase-8, caspase-9 and caspase-12 in PRRSV-infected cells were investigated at 8 h, 12 h, 16 h, 24 h, 36 h and 48 h pi separately (Fig 3), which represents the inductions of extrinsic pathway, mitochondria-dependent pathway, and ER stress-dependent pathway, respectively [52]. The induction of extrinsic pathway leads to the proteolytic processing of procaspase-8, the precursor of caspase-8 [3]. PRRSV-induced cleavage of procaspase-8 was determined by Western blot (Fig 3A). It was shown that STS treatment could induce procaspase-8 cleavage in the uninfected cells compared to the untreated cells. In PRRSV-infected cells, procaspase-8 was significantly reduced from 16 h pi to 48 h pi, indicating the activation of extrinsic pathway at the late stage of infection. An anti-caspase-9 PAb was used to detect both caspase-9 precursor (procaspases-9) and its active form (cleaved caspase-9) (Fig 3B). Upon PRRSV infection, the cleaved caspase-9 was undetectable until 24 h pi, while it became evident at 36 h pi and 48 h pi in comparison with the uninfected cells, and their significance were analyzed by densitometry analysis, suggesting that PRRSV-induced cleavage of caspase-9 is involveed in intrinsic pathway of apoptosis during the late infection. As shown in Fig 3C, cleaved caspse-12 appeared in the presence of TG, an inducer for caspase-12 activation. The level of cleaved caspase-12 was significant starting from 24 h pi (*P*<0.05), and increased prominently at 36 h pi and at 48 h pi (*P*<0.001). Together, these results indicated the involvements of death receptor pathway, mitochondria-dependent pathway, and ER stress-dependent pathway in PRRSV-induced apoptosis in MARC-145 cells.

### Apoptosis Induction in PRRSV-Infected PAMs

**Fig 4:**
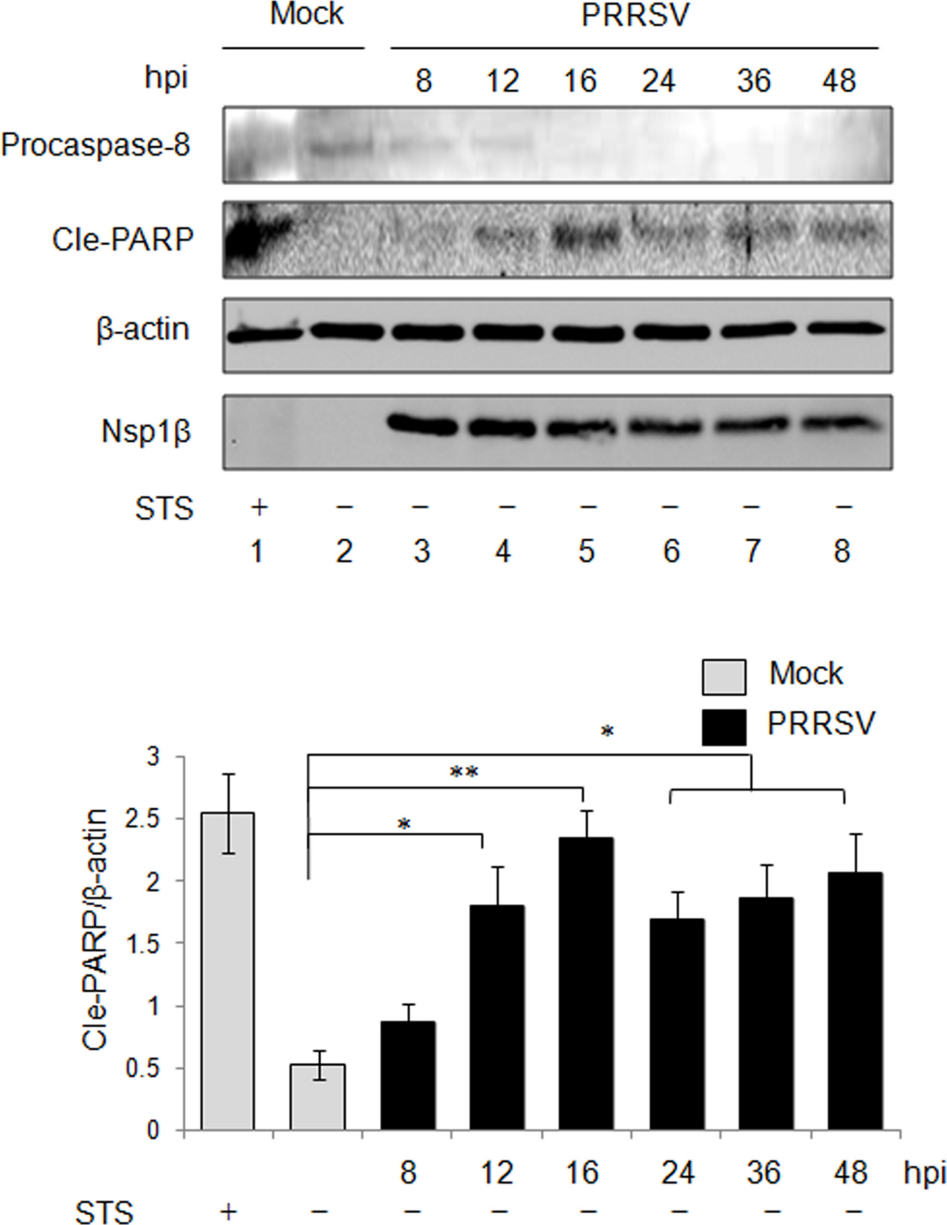
Apoptosis induction in PRRSV infected PAMs. PAMs were infected with PRRSV, and protein samples were harvested at the indicated time points and subjected to Western blot analysis using anti-caspase-8, anti-PARP, and anti-Nsp1β antibodies. β-actin was served as a protein loading control. PRRSV Nsp1β was examined to indicate PRRSV infection. The relative cle-PARP level was analyzed. Significant differences of relative levels between control group and infected groups are indicated (* *P*<0.05; ** *P*<0.01; *** *P*<0.001).

To determine if PRRSV induce apoptosis in PAMs, the principle target cells in PRRSV-infected pigs [53], PAMs were infected with PRRSV for 8 h, 12 h, 16 h, 24 h, 36 h, and 48 h. Cell viability assay showed that the STS treatment had no influence on the viability of PAMs (S1 Fig). The activation of caspase-8 and the cleavage of PARP were examined by Western blot using anti-caspase-8 and anti-PARP antibodies (Fig 4). Virtually, procaspase-8, the caspase-8 precursor, was detected at 8 h pi and 12 h pi, but the procaspase-8 level at these time points were weaker compared to the uninfected control. Procaspase-8 became invisible from 16 h pi to 48 h pi, indicating the activation of caspase-8 initiates at 16 h pi in PRRSV-infected PAMs. The presence of evident cleaved PARP started at 12 h pi, in comparison with the uninfected control, and the increase of cleaved PARP was significant from 12 h pi to 48 h pi (*P*<0.05 or 0.01). The level of cleaved PARP reached a peak at 16 h pi. An anti-Nsp1β antibody was used to detect Nsp1β synthesis in PRRSV-infected PAMs. Overall, our data showed that PRRSV induced apoptosis in PAMs, which is consistent with the findings in MARC-145 cells, whereas the initiation of apoptosis displayed faster in PRRSV-infected PAMs.

### Pro-Apoptotic Functions of PRRSV Nsp4 and Nsp10

**Fig 5:**
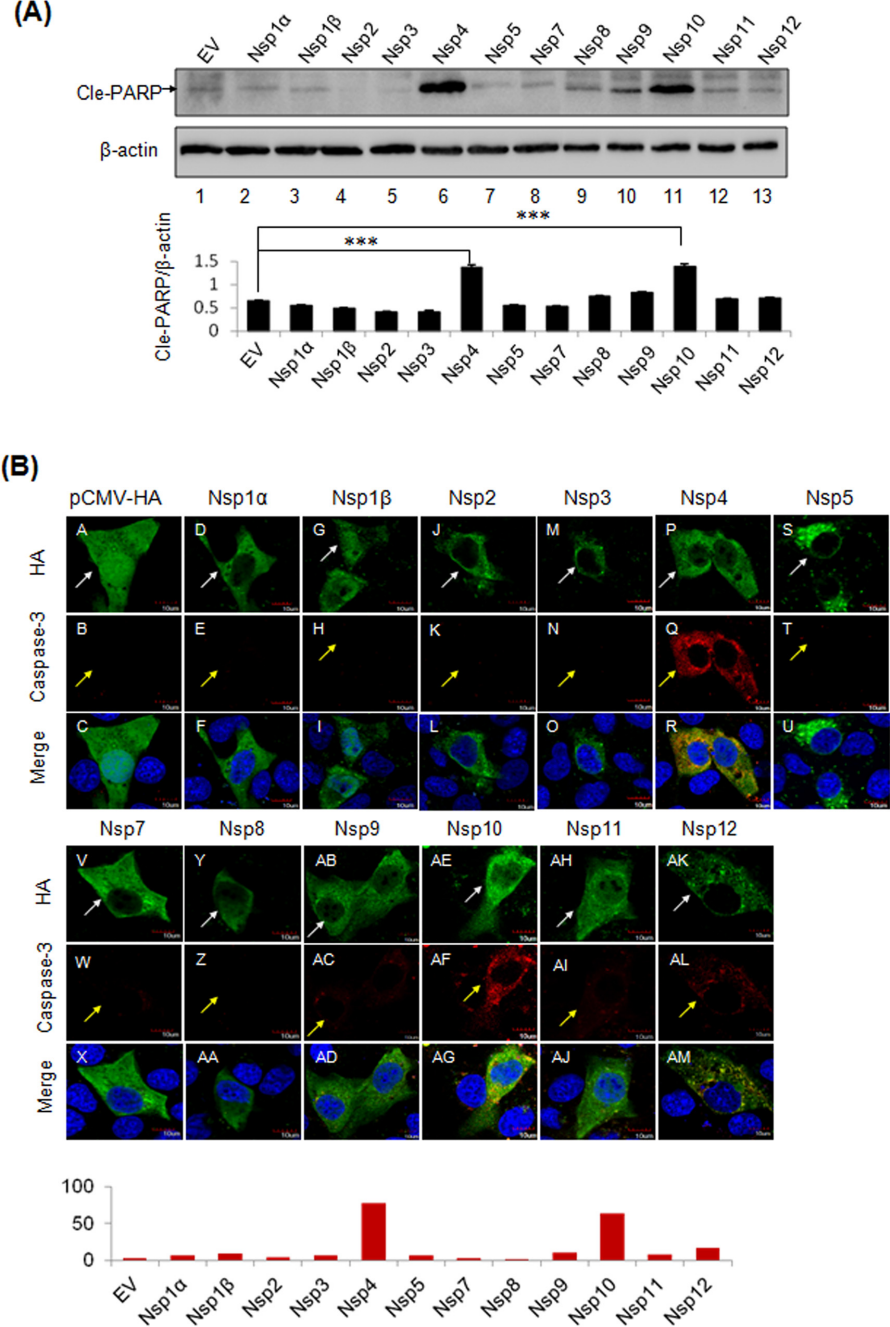
Screening of PRRSV pro-apoptotic inducers. (A) MARC-145 cells were infected with packaged lentiviruses containing individual Nsp of PRRSV, separately. A GFP tag was fused at the C’-terminal of each Nsp. Infected cells were passaged once and cultured for 24 h before harvesting. The cleavage of PARP was examined to determine the induction of apoptosis. β-actin was served as a protein loading control. Empty vector (EV) pWPXL-GFP was used as a negative control. Significant differences of cle-PARP relative levels between EV and PRRSV Nsps were indicated (*P<0.05; ** *P*<0.01; *** *P*<0.001). (B) MARC-145 cells were transfected with DNA constructs that were expressing individual Nsp of PRRSV for 24 h. The cells were fixed and stained with anti-caspase-3 p17 (red, yellow arrows) and anti-HA (green, white arrows) antibodies at 24 h post-transfection. Nucleus was stained by (DAPI) (blue). Scale bars were 10 μm. The average florescence intensities (FIs) of red (caspase-3) in the cytoplasm were analyzed by NIH ImageJ software (lower panel). Red bars indicate the average FIs of red (caspase-3).

The Nsps of PRRSV are multifunctional in virus life cycle [22]**,** and it was plausible that PRRSV Nsps participated in apoptosis induction. The expression of Nsps was achieved using the lentiviruses carrying individual Nsp gene of PRRSV with C’-terminal GFP tag and confirmed under fluorescence microscope (S2 Fig). To screen the viral apoptosis inducers in PRRSV, the cleavage of PARP in MARC-145 cells that were expressing individual Nsp of PRRSV was investigated by Western blot, and IFA was performed to determine the active form of caspase-3 in the cells that were expressing individual PRRSV Nsp using anti-caspase-3 p17 antibody interacting specifically with the cleaved caspase-3 (Fig 5). As shown in Fig 5A, compared to the empty vector (EV) control, cleaved PARP was significantly increased in the cells that were expressing Nsp4 and Nsp10 (*P*<0.001). The expression of Nsp9 induced limited PARP cleavage, which was insignificant in comparison with EV, according to the densitometry analysis. These results indicate the Nsp4 and Nsp10 are pro-apoptotic. Plasmids expressing individual HA-tag conjugated Nsp were constructed and transfected into MARC-145 cells, separately, and each of Nsp was detected by an anti-HA antibody. As shown in Fig 5B, cleaved caspase-3 was dramatically increased in the presence of Nsp4 and Nsp10. By analyzing florescent intensities of red in the cytoplasm, the signal of activated caspase-3 was evident in the presence of Nsp4 and Nsp10, but slightly increased for Nsp9, Nsp11, and Nsp12. Together, our results demonstrated the pro-apoptotic roles of PRRSV Nsp4 and Nsp10 in MARC-145 cells.

### Induction of Apoptotic Pathways by Nsp4 and Nsp10

**Fig 6:**
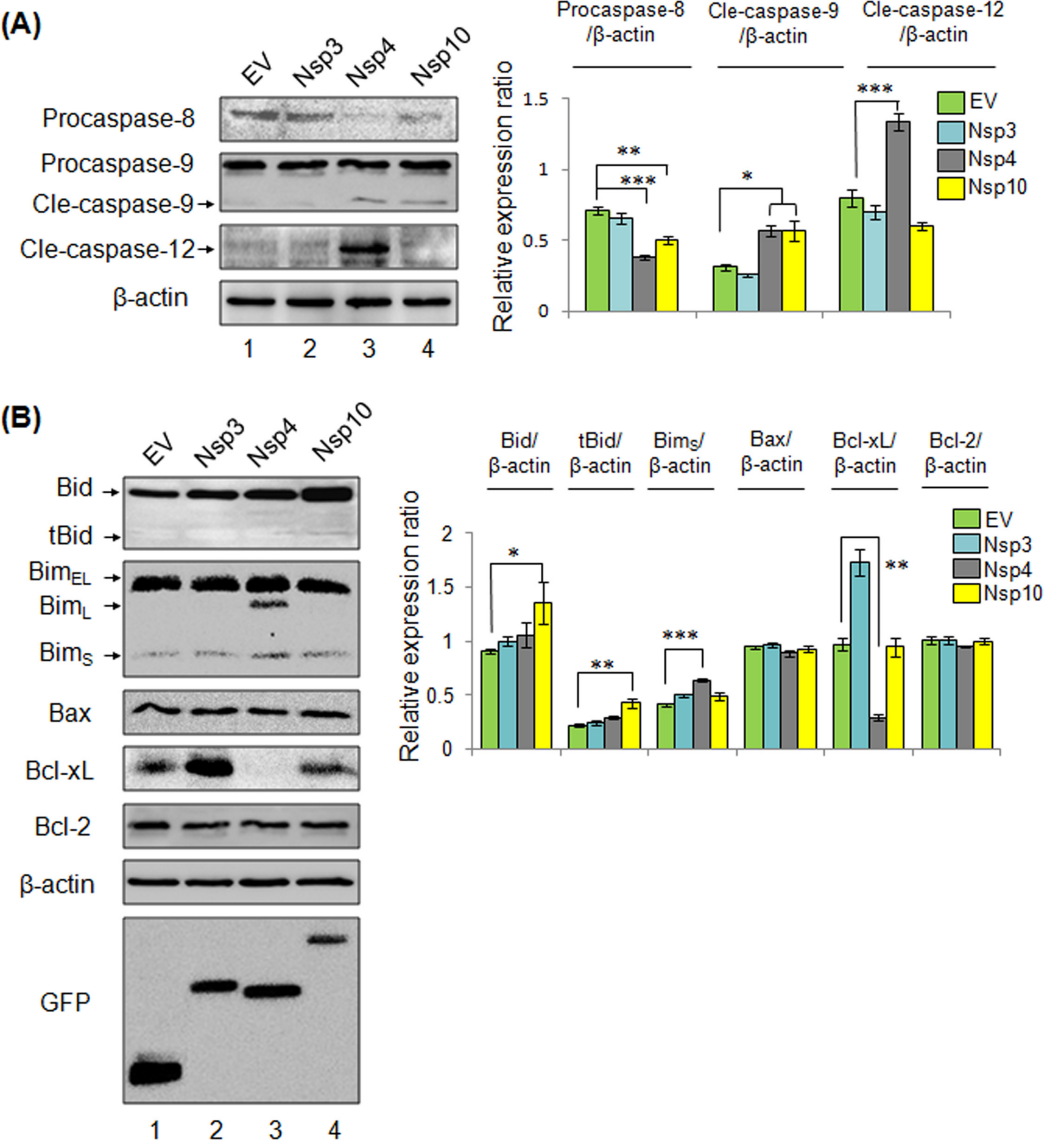
Induction of pro-apoptotic pathways by Nsp4 and Nsp10. (A) Procaspase-8, procaspase-9, and cleved-caspase-12 were examined in MARC-145 cells that were expressing Nsp3, Nsp4, and Nsp10, separately. (B) Protein levels of Bcl-2 family members were examined, including Bid, Bim, Bax, Bcl-xL, and Bcl-2. Black arrows represent each of the indicated isoforms. Empty vector (EV) pWPXL-GFP and Nsp3 were used as negative control. β-actin was used as a protein loading control. Relative proteins levels were calculated for each target protein. Green, blue, grey, and yellow bars represents the cells that were expressing GFP, Nsp3, Nsp4 and Nsp10, respectively.

PRRSV infection was shown to activate death receptor pathway, mitochondria-dependent pathway, and ER stress-dependent pathways (Fig 3). Apoptosis signaling study was further expanded to the cells that were expressing Nsp4 and Nsp10 by examining activation of caspase-8, caspase-9, and caspase-12, and selected Bcl-2 family members were examined by Western blot (Fig 6). As shown in Fig 6A, procaspase-8 was significantly cleaved in the presence of Nsp4 and Nsp10, respectively, compared to Empty vector pWPXL-GFP (EV) and Nsp3 which were served as negative controls. The cleavage of caspase-9 was evident with the expression of Nsp4 and Nsp10, and their cleaved caspase-9 levels were increased significantly compared to EV and Nsp3. These results indicate that both extrinsic pathway and intrinsic pathway are activated by Nsp4 and Nsp10. To identify whether ER stress dependent intrinsic pathway was activated, the activations of capase-12 was determined for the cells that were expressing Nsp4 and Nsp10, separately. The cleavage of caspase-12 was significant in cells expressing Nsp4 but Nsp10 along with EV and Nsp3, suggesting that Nsp4 rather than Nsp10 activates ER stress-dependent pathway.

Bcl-2 family protein members have been proved to participate in PRRSV-mediated apoptosis [33,34,35], it is of interest to investigate if Bcl-2 family members are involved in apoptosis induction by Nsp4 and Nsp10. Bcl-2 family members including Bid, Bim, Bax, Bcl-xL, and Bcl-2 were examined by Western blot. The activation of caspase-8 results in generation of tBid which is pro-apoptotic. As shown in Fig 6B, the expression of Bid was increased significantly with the Nsp10 expression. In addition, tBid appeared only in the presence of Nsp10, whose level was significantly increased compared to EV, Nsp3, and Nsp4. The isoforms of Bim, Bim_L_ and Bim_S_ are pro-apoptotic, which antagonize the anti-apoptotic function of Bcl-xL [54,55,56]. The active isoform Bim_S_ was present in Nsp3, Nsp4, and Nsp10, while Bim_S_ was significantly increased with the Nsp4 expression compared to EV. Isoform Bim_L_ appeared exclusively in the cells that were expressing Nsp4. No significant changes were observed for isoform Bim_EL_ in the presence of Nsps. Besides, no changes of Bax, another pro-apoptotic protein, were observed in the presence of Nsp3, Nsp4, and Nsp10. For anti-apoptotic proteins Bcl-2 and Bcl-xL, Bcl-2 level with the expressions of Nsp3, Nsp4, and Nsp10 were comparable with EV, whereas Bcl-xL was significantly degraded in the presence of Nsp4 compared to the others. These results indicated that Nsp4 was able to manipulate pro- or anti-apoptotic functions of Bcl-2 family proteins to facilitate apoptosis induction.

### Caspase-8 Is Essential for Nsp10-Induced Apoptosis

**Fig 7:**
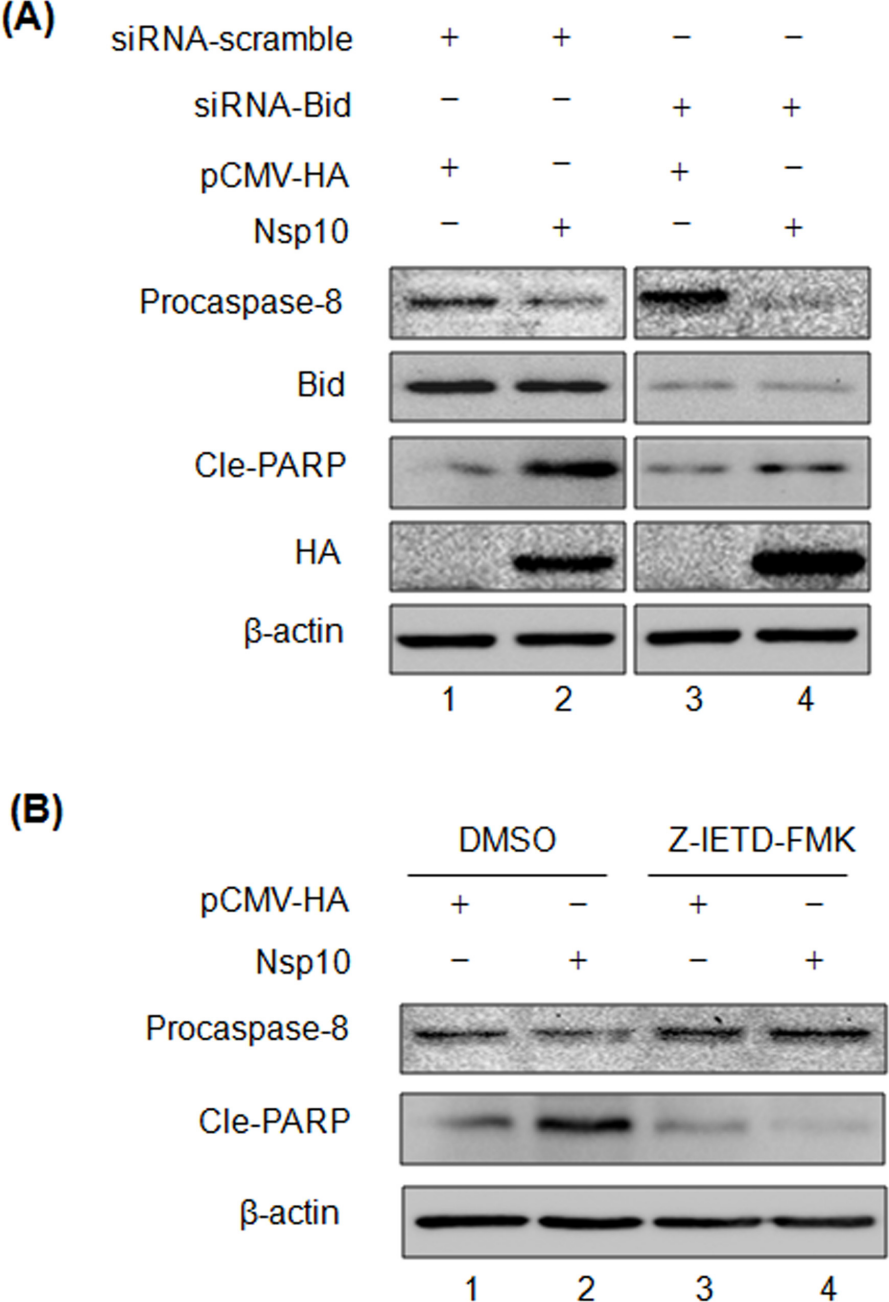
Analysis of an apoptosis signaling crosstalk by Nsp10. (A) Anti-Bid siRNA was transfected into MARC-145 cells or cells that were expressing Nsp10 for 24 h, activation of procaspase-8, Bid expression, and PARP cleavage were examined by Western blot. Anti-HA antibody was used for the detection of Nsp10 expression. β-actin was used as a protein loading control. (B) MARC-145 cells were pretreated with 30 μM Z-IETD-FMK or DMSO for 30 min, and then transfected with the plasmid-expressing Nsp10 or empty vector pCMV-HA for 24 h. Procaspase-8 and PARP were examined by Western blot.

PRRSV Nsp10 was shown to upregulate Bid expression and its cleavage (Fig 6), and Bid has been shown to play a role in PRRSV-induced apoptosis. Thus, we further investigated to see whether Nsp10-mediated apoptosis is depended on the pro-apoptotic function of Bid and whether the pro-apoptotic function of Nsp10 relies on the activation of caspase-8. The siRNA-reduced endogenous Bid expression was evaluated by Western blot, and the effect of caspase-8 activation inhibition on the pro-apoptotic function of Nsp10 was analyzed (Fig 7). The results showed that the endogenous Bid expression was dramatically suppressed in the cells treated with Bid-specific siRNA (Fig 7A) compared to scrambled siRNA-treated cells. Upon the transfection of Bid-specific siRNA, Nsp10-induced PARP cleavage was blocked in comparison with scrambled siRNA-treated Nsp10-expressing cells. These findings suggest that the pro-apoptotic function of Nsp10 is reliant on the pro-apoptotic function of Bid.

PRRSV Nsp10 could activate caspase-8 (Fig 6A), while Bid expression inhibition did not suppress Nsp-10-mediated procaspase-8 activation (Fig 7A). To inhibit the activation of caspase-8, the cells were treated with caspase-8-specific inhibitor, Z-IETD-FMK. As shown in Fig 7B, Z-IETD-FMK treatment blocked caspase-8 activation in the Nsp10-expressing cells compared to the untreated Nsp10-expressing cells. DMSO was used as placebo control, and no inhibitory effect was observed. Furthermore, the cleavage of PARP was evident only in the presence of Nsp10 with DMSO, while Z-IETD-FMK treatment virtually inhibited Nsp10-mediated PARP cleavage, suggesting that caspase-8 activation is essential for Nsp10-induced apoptosis. Overall, these results demonstrated the caspase-8 activation and Bid were indispensable for Nsp10 to induce apoptosis, and Bid might function as signaling linker to establish a crosstalk between the death receptor pathway and mitochondria-dependent pathway.

## Discussion

Virus-infected cells have involved apoptosis in eliminating themselves to prevent viral replication, viral dissemination, or persistent infection [57]. Thus, it is vital for viruses to disable host cell apoptosis at early stage of the infectious cycle, thereby avoiding premature cell death and allowing viral replication. On the other hand, apoptosis can function to promote virus spread at late stage of infection by breaking down infected cells, or killing uninfected cells from the immune system [58]. Recent studies have demonstrated the biphasic apoptosis during virus infection [59,60]. Type 1 PRRSV has been shown to possess a dichotomous apoptotic role in manipulating the apoptosis that the apoptosis is inhibited early in infection but induced late in infection [37]. In the present study, we demonstrated the identical kinetics of apoptosis in type 2 PRRSV-infected cells, suggesting the dichotomous apoptotic role is universal for PRRSV (Figs 1 and 2). Based on the level of cle-PARP, a reliable hallmark of apoptosis, PRRSV was shown to suppress STS-induced apoptosis until 12 h pi in MARC-145 cells, suggesting an anti-apoptotic event at the early stage of PRRSV infection (Fig 1A). Interestingly, the level of cle-PARP appeared to be limited at the time point when the Nsp1β was not detectable and less evident at 4 and 6 h pi (Fig 1A). This finding suggest that small amount of PRRSV-encoded anti-apoptotic products might be sufficient to suppress apoptosis. In addition, PRRSV has been shown to activate PI3K/Akt pathway together with p53 at the early stage of infection, leading to the suppression of apoptosis [35].

In MARC-145 cells, PRRSV could induce apoptosis at 24 h pi with the presence of apparent apoptotic markers (Fig 2). Our results also showed the apoptotic events in the natural host cells of PRRSV, PAMs, suggesting that PRRSV-induced apoptosis is cell type-independent. Compared to MARC-145 cells (24 h pi), an early apoptosis event in PRRSV-infected PAMs at 12 h pi (Fig 4) has been noticed as well. This difference is probably due to the high replication efficiency of PRRSV used in this sudy in PAMs [61]. Previous study has revealed the activations of capsase-8 and caspase-9, indicating that PRRSV can activate both extrinsic apoptotic and intrinsic apoptotic pathways [33]. In this study, we identified the activation of caspase-12 in addition to caspase-8 and caspase-9 in PRRSV-infected cells. Our results suggest PRRSV could induce ER stress-dependent pathways (Fig 3). Our finding is consistent with a previous report that PRRSV activates ER stress with following induction on JNK pathway and apoptosis [35]. Among PRRSV-activated apoptotic signaling pathways, extrinsic pathway is the one with early initiation as the activated caspase-8 appeared at 16 h pi, while the induction of intrinsic pathway and ER stress-dependent pathway started after 24 h pi (Fig 3). Such discrepancy suggests that stimuli for individual apoptotic pathways appear sequentially which relies on the stages of virus life cycle.

PRRSV Nsps encoded by the replicase genes are essential for virus replication, pathogenesis and virulence [22,23]. The roles of Nsps in modulating apoptosis were investigated in this study, and Nsp4 and Nsp10 were proved to function as viral apoptosis inducers (Fig 5). PRRSV Nsp4 belongs to 3C-like serine protease (3CLSP) which mediates the proteolytic processing of polyproteins, pp1a and pp1ab during PRRSV infection [62]. Several viral serine proteases have been proved to induce apoptosis, including severe acute respiratory syndrome-associated coronavirus 3CL^pro^, dengue virus type 2 NS3, and coxsackievirus B3 3Cpro [63–65]. PRRSV Nsp4 was shown to activate caspase-8, caspase-9, and caspase-12, indicating the involvements of extrinsic apoptotic pathway, mitochondria-dependent pathway, and ER stress-dependent pathway (Fig 6). In addition, Nsp4 was able to manipulate the activities of Bcl-2 family members, Bim and Bcl-xL. Bim is a pro-apoptotic protein and processed into three isoforms upon activation, and all of these isoforms are able to promote apoptosis. The isoform Bim_S_ could be detected in the presence of Nsp3, Nsp4, or Nsp10, whereas the isoform Bim_L,_ whose expression is JNK pathway-dependent [66], appeared exclusively in the presence of Nsp4 (Fig 6). Because ER stress activates JNK pathway, and PRRSV Nsp4 was shown to induce ER-stress dependent apoptotic pathway, it is reasonable to suppose that Nsp4 activates Bim indirectly by inducing ER stress. On the contrary, Bcl-xL, an anti-apoptotic protein, was degraded in the cells that were expressing Nsp4 (Fig 6), whose reduction has been proved in PRRSV-infected cells as well [34]. According to a recent study, protease activity of Nsp4 is required for apoptosis induction [41]. Thus, it is possible that Nsp4 is able to cleave Bcl-xL to eliminate its anti-apoptotic activities. However, more molecular details concerning the apoptosis induction by Nsp4 are required to be further done.

PRRSV Nsp10 contains a conserved N’-terminal zinc finger and NTPase/helicase activities resembling with superfamily 1 helicase domain [67]. Nsp10 is believed to be essential for genome replication as a helicase [68], and our recent study has determined that Nsp9- and Nsp10-coding regions of HP-PRRSV are responsible for its replication efficiency in *vitro and in vivo* and fatal virulence for piglets [23]. In the present study, pro-apoptotic function of Nsp10 has been determined, adding a novel function for viral helicase within Nidoviruses. Besides PRRSV, the pro-apoptotic functions of viral helicases are limited to flaviviruses [69–71]. For instance, West Nile virus NS3 can activate caspase-8-dependent apoptosis, and both protease and helicase activities are involved in apoptosis induction. In the cells that were expressing Nsp10, extrinsic and mitochondria-dependent pathways could be induced with the exception of ER stress dependent pathway (Fig 6). Interestingly, activation of caspase-8 has been proved to be indispensable for Nsp10 induced-apoptosis (Fig 7). Nsp10-induced apoptosis also requires the pro-apoptotic functions of Bid protein (Fig 7). Because activated Bid is fully involved in inducting intrinsic apoptotic pathway, it is suggested that Nsp10 can establish a crosstalk between extrinsic and intrinsic pathways. Our results confirmed the active Bid-dependent crosstalk in PRRSV infected cells, consequently, we assume that PRRSV-mediated crosstalk might rely on the pro-apoptotic function of Nsp10 [33], and in this crosstalk, Nsp10 might initiate the apoptosis through the activation of caspase-8, and then the apoptotic signaling could be augmented by Bid. It is essential to further dissect the molecular mechanism in relation to the apoptosis induction by the Nsp 10 of PRRSV.

## Conclusion

In the present study, our results showed the PRRSV-mediated biphasic apoptosis process in which PRRSV infection delays apoptosis at early infection but promotes the apoptosis at late infection. We also determined that the PRRSV-induced apoptosis are involved in the activation of extrinsic pathway, mitochondria-dependent intrinsic pathway, and ER stress-dependent pathway. The Nsp4 and Nsp10 of PRRSV are shown to be apoptosis inducers, and Nsp4 induces apoptosis through manipulations on the pro- or anti-apoptotic functions of Bcl-2 family members, and both activation of caspase-8 and Bid are required for Nsp10-induced apoptosis, suggesting the establishment of a crosstalk between extrinsic- and intrinsic-apoptotic pathways. Our findings add the novel insights into understanding the apoptosis induced by PRRSV and biological functions of PRRSV Nsps.

## Supporting Information

S1 Fig Cell viability assay for the MARC-145 cells treated by STS or TG and PAMs treated by STS. (TIF)

S2 Fig Expressions of PRRSV Nsps in lentiviruses-infected MARC-145 cells. (TIF)

S1 Table Statistical analysis of caspase-3 positive cells in PRRSV-infected MARC-145 cells. (PDF)
