# Supplementary figures and images for: Induction of Apoptosis by the Nonstructural Protein 4 and 10 of Porcine Reproductive and Respiratory Syndrome Virus
Source: PLoS One. 2016 Jun 16;11(6):e0156518. doi: 10.1371/journal.pone.0156518 (PMC4911139; doi:10.1371/journal.pone.0156518)

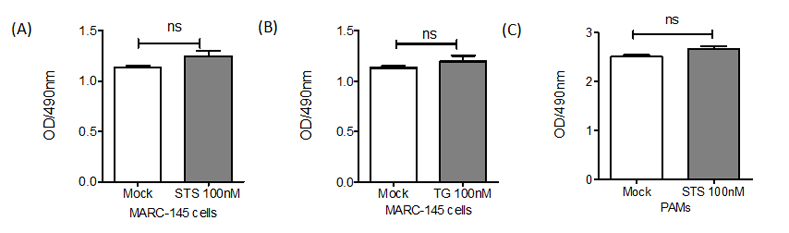

Supplement: S1 Fig — (TIF) [file pone.0156518.s001.tif]

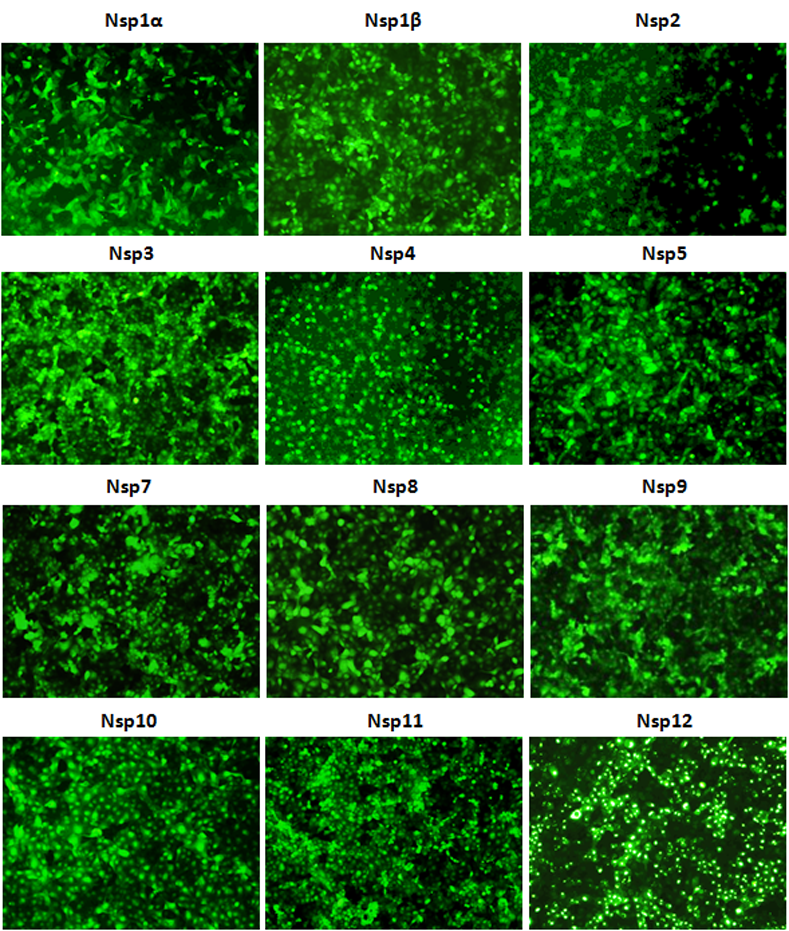

Supplement: S2 Fig — (TIF) [file pone.0156518.s002.tif]
