# Supplementary material for: Induction of Apoptosis by the Nonstructural Protein 4 and 10 of Porcine Reproductive and Respiratory Syndrome Virus
Source: PLoS One. 2016 Jun 16;11(6):e0156518. doi: 10.1371/journal.pone.0156518 (PMC4911139; doi:10.1371/journal.pone.0156518)
Supplement: S1 Table — (PDF) [file pone.0156518.s003.pdf]

S1Table Statistical analysis of caspase-3 positive cells in PRRSV-infected MARC-145 cells

Group	Total cell #	Caspase-3 positive cell												Statistical results			
		Experiment 1				Experiment 2				Experiment 3							
		Gated Cell #	Cas-3 + Cell #	% Cas-3 +	Cas-3 + Ratio (to Mock)	Gated Cell #	Cas-3 + Cell #	% Cas-3 +	Cas-3 + Ratio (to Mock)	Gated Cell #	Cas-3 + Cell #	% Cas-3 +	Cas-3 + Ratio (to Mock)	Ave % Cas-3 +	Ave Cas-3 + Ratio (to Mock)	SD	SD + Error
**Mock**	50000	42622	25	0.06	1.00	46408	36	0.08	1.00	45171	18	0.04	1.00	0.06	1.00	0.00	0.00
**8h**	50000	41500	32	0.08	1.33	45772	75	0.16	2.00	43864	49	0.11	2.80	0.12	2.04	0.60	0.35
**12h**	50000	44818	512	1.14	19.00	45941	1057	2.30	28.75	45927	162	0.35	8.80	1.26	18.85	8.15	4.70
**16h**	50000	42786	250	0.58	9.67	46706	639	1.37	17.13	45028	479	1.06	26.60	1.00	17.80	6.93	4.00
**24h**	50000	37694	6441	17.09	284.83	45113	6463	14.33	179.13	41213	6076	14.74	368.60	15.39	277.52	77.53	44.76
**36h**	50000	31883	26906	84.39	1406.50	41784	29976	71.80	897.50	38194	20128	52.70	1317.50	69.63	1207.17	221.94	128.14
**48h**	50000	24665	21781	88.31	1471.83	30106	24696	82.03	1025.38	22437	13570	60.48	1512.00	76.94	1336.40	220.54	127.33

Cas-3 + stands for Caspase-3 positive. Ave, average. SD, standard deviation.

Cas-3 + Ratio (to Mock) were determined as: the percentage of caspase-3 positive cells of each group/ the percentage of caspase-3 positive cells of the mock group.

PRRSV-infected MARC-145 cells were harvested at the indicated time points, and then fixed and assessed by flow cytometry analysis using direct staining with PE-conjugated active caspase-3 antibody. Totally, 50000 cells were subjected to flow cytometry analysis. The percentage of caspase-3 positive cells and active caspase-3 ratio were calculated for each group. Results of three independent experiments were presented.
